# Using Cyclic Voltammetry, UV-Vis-NIR, and EPR Spectroelectrochemistry to Analyze Organic Compounds

**DOI:** 10.3791/56656

**Published:** 2018-10-18

**Authors:** Sandra Pluczyk, Marharyta Vasylieva, Przemyslaw Data

**Affiliations:** ^1^Faculty of Chemistry, Department of Physical Chemistry and Technology of Polymers, Silesian University of Technology; ^2^Department of Physics, Durham University; ^3^Center of Polymer and Carbon Materials of the Polish Academy of Sciences

**Keywords:** Chemistry, Issue 140, Cyclic Voltammetry, Electron Paramagnetic Resonance, Ultraviolet-Visible and Infra-Red spectroscopy, Organic Electronics, Organic Light Emitting Diodes, Organic photovoltaics, Conjugated Polymers, Electron Affinity, Ionization Potential, Charge Carriers, Spectroelectrochemistry

## Abstract

Cyclic voltammetry (CV) is a technique used in the analysis of organic compounds. When this technique is combined with electron paramagnetic resonance (EPR) or ultraviolet-visible and near-infrared (UV-Vis-NIR) spectroscopies, we obtain useful information such as electron affinity, ionization potential, band-gap energies, the type of charge carriers, and degradation information that can be used to synthesize stable organic electronic devices. In this study, we present electrochemical and spectroelectrochemical methods to analyze the processes occurring in active layers of an organic device as well as the generated charge carriers.

**Figure:**
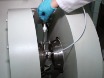


## Introduction

Worldwide, researchers are continually searching for new organic materials that can be used in organic electronics with desirable performance or stability, which drops due to extended use. In the case of organic devices, it is important to understand the behavior of the charge carrier to fully know the rules driving the device behavior. Analysis of the effect of the molecular structure on the generation of the charge carrier and the dynamics and maintenance of the balance of injected charge carriers, both positive (holes) and negative (electrons), is crucial to improve the efficiency and stability of the organic devices. This ensures the effective recombination of these individual charges and consequently significantly improves the photoluminescence efficiency of the organic light emitting diodes (OLEDs)^1^,^2^. For organic photovoltaics (OPVs)^3^,^4^ as well as organic field effect transistors (OFETs)^5^,^6^, it is necessary to have materials with high charge carrier mobility. In addition to the analysis of charge carriers, several important parameters of organic electroactive materials help in predicting where the material could be used: ionization potential (IP), electron affinity (EA) energy levels, and band-gap between them^7^,^8^,^9^,^10^.

In this work, we present a method for the efficient measurement of cyclic voltammetry (CV) that can be used in the analysis of all types of electroactive materials. This technique provides information about redox properties, the doping/dedoping mechanism, the stability, the conversion and storage of energy, *etc*. It also allows for the estimation of electron affinity and ionization energy of the test compounds in a much cheaper and faster way compared to other high vacuum methods. The aforementioned parameters correlate with the energy levels of highest occupied molecular orbital (HOMO) and lowest unoccupied molecular orbital (LUMO).

The method presented in this article can be used to analyze all types of conjugated compounds such as those with delocalized π-electrons in their structures. Conjugated compounds may be small molecules with large polymeric chains. Small molecules can also be monomers; during the initial reaction (photochemical, electrochemical, or chemical) monomers can form polymers. In OLED application, the energy level values are necessary to enable the use of the correct host for the emitter in a thermally activated delayed fluorescence (TADF) guest-host system or to decide with which compounds the exciplex donor-acceptor layer could be formed and what additional layers (electron transporting layer (ETL), hole transporting layer (HTL), electron blocking layer (EBL), and hole blocking layer (HBL)) will be necessary to synthesize stable efficiently charged balanced OLED devices^11^,^12^,^13^,^14^,^15^,^16^,^17^. Additional electrochemical measurements allow the investigation of possible side reactions during the process of degradation of the active layer and the formation of low mobile charge carriers (bipolarons)^18^,^19^,^20^,^21^,^22^.

Coupling electrochemical and spectroelectrochemical methods allows for easy, accurate, and reliable determination of the degree of oxidation or reduction of conjugated compounds and their degradation potential, which is crucial for stability^23^,^24^,^25^,^26^,^27^,^28^. Ultraviolet-visible and near-infrared (UV-Vis-NIR) spectroscopy coupled with electrochemistry can characterize the fundamental chromatic properties of all new conjugated compounds, such as the changing of the absorption band during doping^18^,^19^,^20^,^21^,^22^,^23^,^24^,^25^,^26^,^27^,^28^,^29^,^30^.

In a study related to the doping mechanism, it is important to define the type of charge carriers. In this process, two classes of charged quasiparticles take part, one with uncompensated spin (polarons) and the second being diamagnetic (bipolarons); electron paramagnetic resonance (EPR) spectroscopy provides invaluable assistance, which directly allows one to observe and track changes in populations of paramagnetic polarons^29^,^30^,^31^,^32^. In small molecules, it is difficult to form bipolarons, but these molecules can be quite conjugated and have bipolaron-inducing properties; it is important to check if and at which potential polarons and bipolarons are formed in the structure. Bipolarons are at least one order lower in mobility than that of polarons; therefore, if bipolarons are formed in working devices, then it could lead to an unbalanced ratio of the charge carriers, which would result in high current and overheating of the OLED device or may well be the centers of degradation^33^.

The method of measurement proposed in this study is cheap and faster and allows for the determination of the most valuable operative parameters for a large number of electroactive materials without the need for special devices that are based on newly synthesized materials to check its performance. By applying electrochemistry and spectroelectrochemistry, it is possible to select one material that is really promising from hundreds of new materials. In addition, it is possible to obtain detailed information regarding the processes of doping and their effects on the chemical structure of the test conjugated systems using electrochemical and spectroelectrochemical methods, which allows constructing more efficient organic electronics devices.

## Protocol

### 1. Preparation of the Experiment

1. Prepare 25 mL of 0.1 M electrolyte solution. NOTE: Depending on the compounds under investigation, use different electrolytes as the mixture of organic salt and solvent: the most common salts used in the analysis of organic molecules are tetrabutylammonium hexafluorophosphate (Bu_4_NPF_6_) or tetrabutylammonium tetrafluoroborate (Bu_4_NBF_4_); the most common solvent used in the analysis are dichloromethane, acetonitrile, or tetrahydrofuran.
  1. Choose the electrolyte solvent based on the solubility of the test compounds. It should be a solution during investigation but be in a solid state when the material is used in the making of the device, as a thin film deposited on the working electrode surface.
2. Clean the electrodes to be used in the experiment^29^.
  1. For electrochemical measurements, use a 1 mm diameter platinum disk electrode as the working electrode (WE), a platinum coil or wire as the auxiliary electrode (AE), and a silver/silver chloride (Ag/AgCl) electrode as the reference electrode (RE).
  2. For EPR spectroelectrochemical measurements, use platinum wire as the WE, a platinum coil or wire as the AE, and Ag/AgCl as the RE.
  3. For UV-Vis-NIR spectroelectrochemical measurements, use a quartz indium tin oxide (ITO) or fluorine-doped tin oxide (FTO) as the WE, a platinum wire as the AE, and an Ag/AgCl electrode as the RE.
  4. Polish the platinum disk electrode by rubbing the downside of the electrode (the platinum electrode working area) on a polishing pad covered with 1 µm alumina slurry for 3 min.
    1. Rinse the electrode with deionized water to remove all of the slurries from the electrode and clean in an ultrasound bath (320 W, 37 kHz) with deionized water for 15 min.
    2. Rinse the electrode using a 1 mL syringe with isopropanol (3 x 1 mL) and then with acetone (3 x 1 mL).
    3. Use a paper towel to remove all solid residues and dry in the air for 3 min.
  5. Clean the ITO and FTO electrodes with deionized water. Place them in an ultrasound bath (320 W, 37 kHz) in acetone for 15 min and then in isopropanol for the next 15 min.
  6. Burn the platinum electrodes, wires, and coils using a high-temperature gas torch (>1000 °C) for 1 min. Be careful! Use tweezers to hold the electrodes. Wait for 5 min after burning and before using the electrodes.
3. Clean the electrochemical and spectroelectrochemical cells (**Figure 1**) with water and then with acetone. Use a paper towel to remove all the solid residues. Clean all other elements (ca. PTFE parts) with acetone and dry in the air before use.

### 2. CV Analysis

1. Turn on the potentiostat and computer.
2. Fill an electrochemical cell with 1.5 mL of the electrolyte solution to be analyzed.
3. Put all the three electrodes (WE, AE, and RE) in a cell (the cell’s cap is equipped with the PTFE electrode holder, so put electrodes through the holes in this holder) and connect it to a potentiostat. Keep the WE and RE as close to each other as possible (**Figure 1**). Follow the information regarding the connection of each wire of the potentiostat to its respective electrode.
4. If needed (for example during reduction analysis), put the argon (or nitrogen) pipe (through an additional hole in the electrode holder) and start bubbling the solution for at least 5 min. After this, move the argon (or nitrogen) pipe above the solution’s level and keep the gas flow running for the measurement. NOTE: The cell looks closed but cannot be completely leak-proof; therefore, a method to remove the redundant amount of gas from the cell must be available (*e.g*., by an additional small hole in the electrode holder). The pressure depends on the pipe used; set the pressure to high as it is required to set the slow gas flow. If a highly volatile solvent is used in the experiment (*e.g.,* dichloromethane) or the experiment is taking a long time (more than 30 min), then use the Drechsel bottle to saturate argon (or nitrogen) gas with the solvent used in the measurement.
5. Run the potentiostat software, choose the CV procedure and use the following settings: start potential of 0.00 V, upper vertex potential of 2.0 V, lower vertex potential of 0.00 V (if investigating the process of reduction, then a lower vertex potential of −2.5 V), stop potential of 0.00 V, number of stop crossing of 6, and scan rate of 0.05 V/s.
  1. In the section **Export ASCII data**, define a **filename** and choose a folder to save the data in, and push the **START** button (decrease or increase the lower and upper vertex potential to fit the electrochemical window or the sample electrochemical activity)
6. If any peaks are visible in the positive range of potential, then repeat the cleaning procedure. If there is a peak in the negative range potential, then put the argon (or nitrogen) pipe in the solution and start bubbling for an additional 5 min.
7. Add a drop of 1 mM ferrocene (in the solvent used to prepare the electrolyte) to the electrolyte solution by using a syringe.
8. Set the start potential to 0.00 V, the upper vertex potential to 0.85 V, the lower vertex potential to 0.00 V, the stop potential to 0.00 V, the number of stop crossing to 10, and the scan rate to 0.05 V/s. In the section **Export ASCII data**, change the **filename** and push the **START** button.
9. After the measurement, finish by repeating the cleaning procedures as mentioned in steps 1.2 and 1.3.
10. Prepare 4 mL of 1 mM test compound in the prepared electrolyte (step 1.1).
11. Fill the electrochemical cell with the test compound solution (1.5 mL); put all three electrodes in a cell and connect to the potentiostat (step 2.4). To investigate the process of reduction, remove the oxygen (step 2.5).
12. Set the start potential to 0.00 V, the upper vertex potential to 0.50 V (or 0.00 V in case of investigation of reduction process), the lower vertex potential to 0.00 V (−0.50 V in case of investigation of reduction process), the stop potential to 0.00 V, the number of stop crossing to 10, and the scan rate to 0.05 V. In the section **Export ASCII data**, change the **filename** and push the **START** button.
13. Repeat step 2.13 by increasing upper vertex potentials (or by decreasing the lower vertex potential) by 0.1 V until peak registration (**Figure 2a**). If the successive scan is shifted in potential (**Figure 2b**), then clean the RE and leave it in the electrolyte solution. Then, repeat the measurement.
14. Measure both oxidation and reduction processes on the same CV for the determination of IP and EA: set the start potential to 0.00 V, the upper vertex potential to 1.00 V, the lower vertex potential to −2.70 V, and the stop potential to 0.00 V. Choose the upper and lower vertex potential in a way that registers full reduction and oxidation peaks and avoids further redox steps (if any exists) (**Figure 2a**). NOTE: Estimate IP and EA by measuring the onset potential. There are a couple of ways to calculate these parameters from CV but the most useful is to calculate using the onset potential. Sometimes, it is not possible to observe reversible redox couple, especially for both *n* and *p* doping. Mixing of two different techniques is also undesirable as it may provide wrong results and conclusions.
  1. To measure the onset potential, mark the tangent line to the CV peak (**Figure 3**). Calculate the desired values from the onset potential of the reduction or oxidation process using equations (**Equations 1 and 2**)^10^,^18^,^19^,^36^,^37^,^38^. 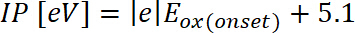                              (1) where 
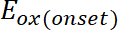
 is the onset of the oxidation potential calibrated with ferrocene^*^ 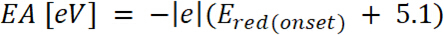                              (2) where 
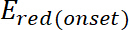
 is the onset of the reduction potential calibrated with ferrocene^* ^^*^Potential calibrated with ferrocene means that the value of potential from the measurement is reduced by the oxidation potential of ferrocene.
15. Pull out the electrodes and pour out the test solution into the proper waste container.
16. Repeat the cleaning procedure (steps 1.2 and 1.3).
17. Repeat steps 2.9–2.12 with 0.25, 0.10, and 0.20 V/s scan rates.
18. Add one drop of 1 mM ferrocene (in the solvent used to prepare the electrolyte solution) to the test solution using a syringe. Set the start potential to 0.00 V, the upper vertex potential to 0.60 V, the lower vertex potential to 0.00, the stop potential to 0.00 V, the number of stop crossing to 6, and the scan rate to 0.05 V. Push the **START** button. After the measurement is completed, change the upper vertex potential to a value that registers the ferrocene couple with the first peak of oxidation of the test compound.
19. Pull out the electrodes and pour out the test solution into the proper waste container.
20. If the electropolymerization occurs during electrochemical oxidation, wash the WE carefully with the electrolyte solution using the syringe (3 ´ 0.5 mL). Clean the RE, AE, and electrochemical cell using the standard procedure (steps 1.2-1.3).
21. To investigate electrochemical products deposited on WE, proceed to step 2.23. To investigate a solution, proceed to step 1.2.1 and start again from step 2.3.
22. Fill the electrochemical cell with an electrolyte solution. Then, put all the three electrodes in a cell and connect them to a potentiostat (step 2.4). To investigate the reduction process, deoxidate the solution (step 2.5).
23. Repeat steps 2.10–2.12 and then step 2.16.

### 3. UV-Vis-NIR Spectroelectrochemical Analysis

1. Measurement preparation
  1. Turn on the spectrometer and potentiostat.
  2. Fill the spectroelectrochemical cell with 0.5 mL of electrolyte solution (step 1.1).
  3. Put the PTFE seal on one side of the cell; then put the ITO electrode to the cell in this way to have a conductive side of the electrode touching the PTFE seal. Put the rest of the PTFE parts as shown in **Figure 1**. Put the RE and AE through the PTFE electrode holder and cover the upper side of the ITO electrode with copper foil as shown in **Figure 1 **for better conductivity.
    1. Put the assembled cell in the holder of the spectrometer and connect all the electrodes to a potentiostat. Follow the information regarding the connection of each wire of the potentiostat to its respective electrode.
  4. Run the potentiostat and spectrometer software.
  5. In the spectrometer software, choose **File | New | Absorbance** measurement.
  6. Choose one of the listed detectors.
  7. Make sure that the button on the detectors is in the “open” position. Then, click on the glowing light bulb; close the detector (move the button to the closed position) and then, click on the dark light bulb.
  8. Select the second detector and repeat step 3.1.7.
  9. Reconnect electrodes from the potentiostat and pull out the electrodes and other elements of the spectroelectrochemical cell.
  10. Repeat cleaning procedures (steps 1.2 and 1.3).
  11. Fill the spectroelectrochemical cell with a diluted (1´10^−5^ M) solution of the test compound in the electrolyte solution if testing the small molecule. Fill the spectroelectrochemical cell with the electrolyte if testing the polymeric (oligomeric) layer deposited on the ITO electrode.
  12. Assemble the spectroelectrochemical cell (step 3.1.3).
  13. Click **Save**.
  14. Select one of the listed detectors.
  15. Define the save location and name the file (include the selected detector).
  16. Select the second detector and repeat step 3.1.15.
2. Potentiostatic Analysis^18^.
  1. Apply a neutral potential (*i.e.,* 0.00 V). This spectrum will be the starting spectra. Increase potential by 0.1 V and wait ca. 10 s until the process stabilizes and records absorption spectra (steps 3.1.13–3.1.16). Continue the measurement to the first change in UV-Vis-NIR spectra; wait for 10 s and then save the spectra (steps 3.1.13–3.1.16).
  2. Increase potentials by 0.05 V; wait for 10 s and save (steps 3.1.13–3.1.16).
  3. Repeat 3.2.2 until achieving the potential of the first or second oxidation potential (these potentials are known from the CV measurement); then, reverse the potential and go back to the starting potential.
3. When the measurements are completed, measure the CV of the test solution. Add 1 mM ferrocene (10 μL) and measure the CV again. The value of ferrocene will help estimate the potential of the process and unify the data.
4. This analysis provides information regarding the optical band-gap, maximum wavelength of undoped and doped states, and isosbestic points of the electrochemical process. Calculate the optical band-gaps from onset values of π-π^*^ bands on the UV-Vis spectra and using **Equation** **3**^18^,^19^,^23^,^24^. 
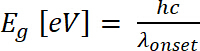
                                              (3) where *h* is Planck’s constant, *λ_onset_* is compound absorption onset,* and c* is the speed of light in vacuum.
5. Time-Resolved Potentistatic Analysis^19^,^32^,^33^
  1. Prepare the measurement similar to the method described in 3.1.1–3.1.16 and put the argon (or nitrogen) pipe (through an additional hole in the electrode holder) and bubbling the solution at least 5 min prior to the measurement. After this, move the argon pipe above the solution level and keep the gas flow running for the measurement.
  2. Run the potentiostat software, choose the chronoamperometry procedure with the following settings: potential start potential to 0.00 V, upper potential to 1.0 V, lower potential to −1.00 V, duration as 5 s, interval time as 0.010 s, number of repeats to 10, and delay time to 10 s. In the section **Export ASCII data**, define a **filename** and choose a folder to save the data.
  3. In the spectrometer software, choose **File | New | High-speed Acquisition**. A new window will appear; set up the following parameters: Integration Time—10 ms, Scans to Average—1, Boxcar Wirth—0, Number of Scans—10000. Do not click the **Go** button.
  4. In the potentiostat software, push the **START** button. When the countdown drops to 1 s, press the **Go** button in the spectrometer software.
  5. Note that it is not possible to see absorption results until the process finishes.
  6. Analyze the data to provide parameters. Use the information saved in the file such transmittance, current, voltage, and current density to provide working parameters:
    1. Calculate Optical Density (ΔOD), the absorbance of the test compound during doping (dedoping) with **Equation 4**: 
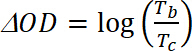
                               (4) where *T_b_* is transmittance in doped form, a “bleach” state [%], and *T_c_* is transmittance in undoped form, a “colored” state [%].
    2. Calculate Coloration Efficiency (CE, η), the amount of electrochromic color formed by the used amount of charge and as the connection between the injected/ejected charge as a function of electrode area (Q_d_) and the change in the optical density (ΔOD) value at a specific wavelength (λ_max_) with **Equation 5**. Its value depends on the wavelength chosen for study, so the value is measured at λ_max_ of the optical absorption band of the colored state. 
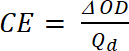
                               (5) where *ΔOD* is the optical density [-] and *Q_d_* is the charge density [C/cm^2^].
    3. Calculate the Contrast Ratio (CR), the measured intensity of color change during electrochemical doping and usually characterized by λ_max_ of the colored form with **Equation 6:** 
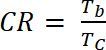
                               (6) where *T_b_* is transmittance in doped form, “bleach” state [%], and *T_c_* is transmittance in undoped form, “colored” state [%].

### 4. EPR Spectroelectrochemical Analysis

1. Measurement preparation
  1. Turn on the spectrometer and the potentiostat.
  2. Fill the spectroelectrochemical cell with electrolyte (step 1.1).
  3. Set the power to 1 mW and tune the resonator to achieve a sharp, centered signal in Q-dip (use the autotuned option).
  4. Set the Mn marker (manganese standard) to 600 and close the Q-dip window. If there is no Mn marker, then skip this step and go to step 4.8.
  5. Set “sweep width ±” to 2.5´ 10 mT, “mod width ±” to 1.0´0.1 mT, “amplitude” to 5´100, “time constant” to 0.03 s, and “center field” value to *ca.* 338 mT and start the measurement.
  6. If not registering six spectral lines of Mn marker, then change the “center field” value.
  7. When registering six spectral lines of Mn marker (**Figure 4**), set the “center field” value between third and fourth line and decrease the value of “sweep width ±” to a value that covers only these two lines. Start the measurement again.
  8. Go into the Q-Dip option, set the Mn marker to 0, and close the Q-dip window.
  9. Measure the pure electrolyte as the background and check if there are any signals. If the signals are visible, then the signal may be from the contamination, low oxidation potential compounds, or from the glass.
  10. Clean the spectroelectrochemical cell (step 1.3).
2. Investigation of small molecules (solutions).
  1. Fill the spectroelectrochemical cell with a diluted (1´10^−5^ M) solution of the test compound in the electrolyte; put all three electrodes through the electrode holder to the cell in this manner so that the WE and RE are inside the spiral AE (**Figure 1**).
    1. Put the WE close to the bottom of the cell and the RE at the upper side of the active (metal not covered by PTFE) part of the WE (**Figure 1**). Place the cell equipped with an electrode inside in the sample cavity of the spectrometer and connect electrodes with a potentiostat (follow the information regarding the connection of each wire of the potentiostat to its respective electrode).
  2. Apply the potential corresponding to the onset of the first redox peak (value known from CV measurement). If a signal appears, then the equipment is set up properly.
  3. Go into the Q-Dip option, set the Mn marker to 600, close the Q-dip window, and start the measurement. The registration of signal with the signal of Mn marker allows calculation of the *g*-factor of the received signal.
  4. Go into the Q-Dip option, set the Mn marker to 0, close the Q-dip window, and wait *ca. *5 min.
  5. Set the “center field” value in the center of the signal, decrease the value of “sweep width ±” to a value that covers the signal, but does not cut off the beginning and ending of the signal (when “sweep width ±” is changed, check entire signal), and decrease the value of “mod width ±” to get well-resolved spectra.
  6. If the signal is small, then increase the “amplitude” value and “time” (acquisition time) of the measurement.
  7. To decrease the signal to noise ratio, set the acquisition to 4, 9, or 16 depending on how noisy the signal is. If the signal is very noisy, select 16.
3. Investigations of the polymeric layer deposited on the surface of the WE.
  1. Fill the spectroelectrochemical cell with an electrolyte solution (step 1.1).
  2. Put electrodes into the cell. Be careful not to destroy the polymeric layer on the WE. Connect the electrode with the potentiostat (step 4.2.1).
  3. Apply neutral potential (*i.e.* 0.00 V) to ensure that the compound is in a neutral state; this spectrum will be the starting EPR spectra.
  4. Increase potential by 0.10 V. Wait *ca.* 10 s until the process stabilizes and record the EPR spectra.
  5. Repeat step 4.3.4 when the EPR signal appears.
  6. Increase the potential by 0.05 V. Wait *ca.* 10 s until the process stabilizes and record the EPR spectra.
  7. Repeat step 4.3.6 until the first or second oxidation potential is achieved and so on (values of these potentials are known from CV measurement); then, reverse the potential and go back to starting potential.
  8. Apply the potential at which the EPR signal appeared in the previous cycle (4.3.5), go into the Q-Dip option, set the Mn marker to 600, close the Q-dip window, and start the measurement. Register the signal with the third and fourth spectral line of Mn marker (4.1.7).
4. When the measurement is completed, analyze the data.
5. Some of the analysis such as a number of spins can be only performed for the compounds deposited (insoluble polymers) on the WE. To calculate the number of spins, double-integrate the EPR signal, followed by a comparison of obtained value with calibrated Mn internal standard. Calculate the number of repeating units forming the polymer on the electrode using **Equation 7**^24^,^32^,^33^: 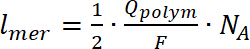                               (7) where *l_mer_* – number of repeating units in the polymer, *Q_polym_* is the polymerization charge, *F* is the Faraday constant, and *N_A_* is the Avogadro number.
6. To calculate doping charge, use the CV of deposited polymer recorded in a spectroelectrochemical cell before the EPR measurement, using **Equation 8**: 
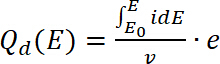
                               (8) where *Q_d_* is the doping charge, *i* is the current, *E* is the set potential, *E_0_* is the starting potential, *v* is the scan rate, and *e* is the elemental charge.
7. Use calculated doping charge to calculate doping level, using **Equation 9**: 
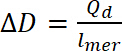
                                              (9) where *∆D*- doping level, *Q_d_* is the doping charge, and *l_mer_* – number of repeating units in the polymer
8. To calculate the number of bipolarons, assume an initial doping level equal to zero. Use the number of polarons per unit from the concentration of spins, so the number of bipolarons should be calculated using **Equation 10. **In the calculations, assume that the initial amount of bipolarons is zero and that faradaic current is much higher than that of non-faradaic current. 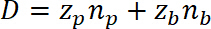                               (10) where *z_i_* is the charge carrier’s elemental charge, *n_i_*is the number of charge carriers per polymer unit, (*p*) is the polarons, and (*b*) is the bipolarons.
9. Extract the *g*-factor value from the EPR spectra. Determine using Mn as an internal standard (steps 4.2.3 and 4.3.8), knowing that the fourth manganese line in it has a *g*-factor of 2.03324. Calculate the line width of EPR signal as the distance in mT between minimum and maximum of the EPR signal. This value is important as it may tell where (on which atom) the radicals are formed.

## Representative Results

The most common application of CV analysis is the estimation of IP and EA. Even though there are a couple ways to obtain CV data, it is strongly recommended to calculate them based on the onset of redox peaks (Figure 3). This approach allows unifying the calculation procedure. Not all of the tested materials undergo reversible oxidation/reduction processes (Figure 3); in such situations, it is not possible to calculate based on average potentials (average from potentials correspond to a maximum of cathodic-reduction and anodic-oxidation peaks). However, it is almost always possible to run the tangent to a peak as shown in Figure 3. With the intersection with the background line and use of **Equations 1 and 2**, the IP and EA values are estimated as 5.35 eV and −2.90 eV, respectively. There are also several different scales used to evaluate IP and EA based on CV measurements. The most commonly used scales for organic materials are −4.8 and −5.1 eV as equivalent to 0.00 V versus Normal Hydrogen Electrode (NHE). However, all of the scales are only an approximation; remember this when comparing different results. The crucial thing is to state what parameters were considered for calculations. In this case, the value −5.1 eV has been chosen as it should correspond to the formal potential of the ferrocene redox couple; in the Fermi scale, it is 0.40 V versus Saturated Calomel Electrode (SCE) in acetonitrile, which is in agreement with the previous measurement.

There are many articles published regarding the analysis of CV. Herein, we show when the process is not going to be as it is expected. The analysis is based on thiophene derivative: NtVTh (structure shown in Figure 4), which undergoes degradation upon oxidation (Figure 5).

NtVTh has two oxidation potentials: the first at 0.70 V and the second at 0.84 V (Figure 5). During the first cycle, the reduction peak is not observed, indicating an irreversible process. Electrochemical characteristics of NtVTh show that polymerization does not occur and after the first oxidation potential, some electro-inactive layer of reaction products deposits at the electrode surface, thus hindering the polymerization process. What is visible is the reaction with the radical cation on the vinyl bond, where the molecule is losing its conjugation and form dimers on the electrode.

While it is difficult to extract information about charge carriers from the CV, it is possible to distinguish between polarons and bipolarons when supported by a UV-Vis-NIR spectrometer. The neutral poly(O*i*PrThEE) was characterized by two wide absorption π|π* transitions bands with peaks maxima at λ_max1_ = 363 nm and λ_max2_ = 488 nm, related to the aromatic form of the undoped polythiophene derivative. During the oxidative doping, new polaronic and bipolaronic bands are generated. The UV-Vis spectrum obtained during the poly(O*i*PrThEE) oxidation revealed the diminishing the neutral polymer absorption band (300-550 nm) (Figure 6) together with the formation of a new absorption band (550-950 nm) of the radical cations of bithiophene and *p*-phenylenevinylene with maxima at 692 nm. The isosbestic point of the oxidation process was located at 604 nm. The bipolaronic band appeared between the 950 nm and 1700 nm with a maximum located at 1438 nm.

EPR spectroscopy is the technique that detects materials with an unpaired electron, this includes organic radicals^39^. There are several parameters that could be extracted from EPR spectra, but one of the most interesting is to estimate where the radicals are localized. Electrons, similar to protons, possess spin. By placing an electron in an external magnetic field, this spin can be split two ways: parallel and antiparallel to the magnetic field, giving two energy levels. This phenomenon is known as the Zeeman effect^40^,^41^. In case of organic radicals, the unpaired electron interacts not only with the external magnetic field but also with magnetic nuclei (nuclei which have a nonzero spin; I≠0). A number of degenerate energy levels are equal to 2I + 1, where I is the spin quantum number of the nucleus with which the unpaired electron interacts^42^. The interaction of the unpaired electron with a larger number of magnetic nuclei leads to further splitting of the energy levels and to hyperfine structure of EPR spectra registration^43^ (Figure 7).

For molecules where the unpaired electron interacts with an even larger number of nuclei, the individual spectral line could overlap, which results in registration of a single, broad signal^44^,^45^,^46^ (Figure 8). This is typical for conjugated polymers, where the generated radical ion during a redox process is delocalized^47^,^48^.

The combination of EPR spectroscopy with electrochemical methods allows the characterization of charge carriers (radical ion) generated during the redox process as well as the determination of the mechanism of these processes^49^,^50^. If well-resolved (peaks are separated; not in the form of one broad peak) spectra are registered, as in the case of electrochemical reduction of *s-*tetrazine derivative (Figure 7), then the analysis of the hyperfine structure of spectra leads to conclusions about the localization of unpaired electron. One way to analyze this kind of spectra is to conduct simulation with special software and to fit simulated spectra with the experimental one^51^. This is especially helpful when the hyperfine structure is complex due to the interaction of the unpaired electron with large numbers of protons. In case of the *s*-tetrazine derivative shown in Figure 7, simulation of EPR spectra (red line) indicates the interaction of the unpaired electron with four nitrogen atoms of *s*-tetrazine.

**Figure:**
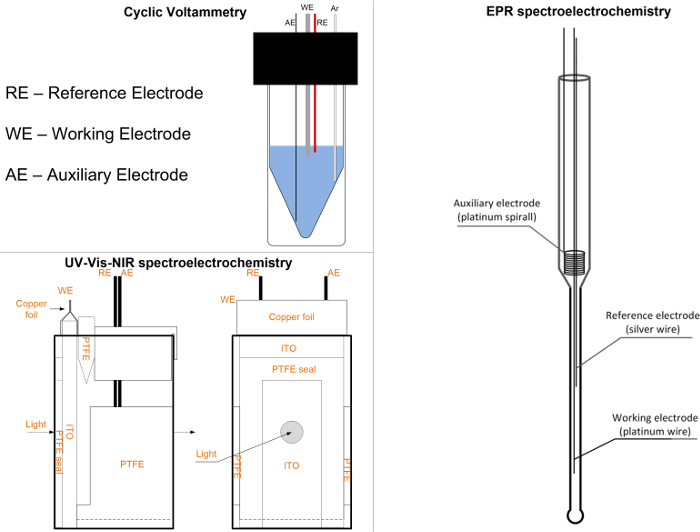

**Figure 1: Electrochemical and spectroelectrochemical cells used for measurements.** The figure presents the scheme setup of electrochemical/spectroelectrochemical cells using cyclic voltammetry, ultraviolet-visible and near-infrared (UV-Vis-NIR), and electron paramagnetic resonance (EPR) spectroelectrochemical measurements. Please click here to view a larger version of this figure.

**Figure:**
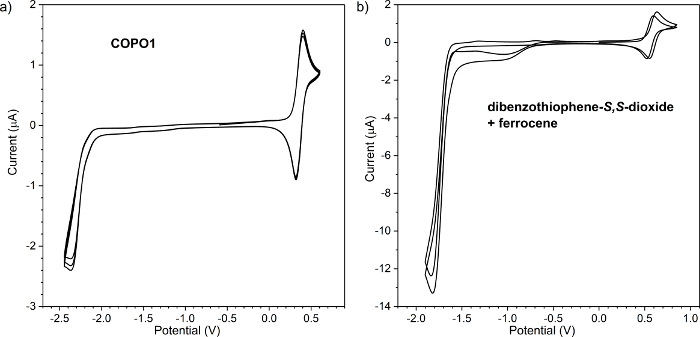

**Figure 2: Cyclic voltammetry (CV) of properly measured compound COPO1 (a) and the CV with an unstable reference potential dibenzothiophene-*S,S*-dioxide with ferrocene (b)^52^. **The figure shows two cyclic voltammograms. (a) presents correctly registered CV and (b) shows a voltammogram registered using a reference electrode with no stable potential. Please click here to view a larger version of this figure.

**Figure:**
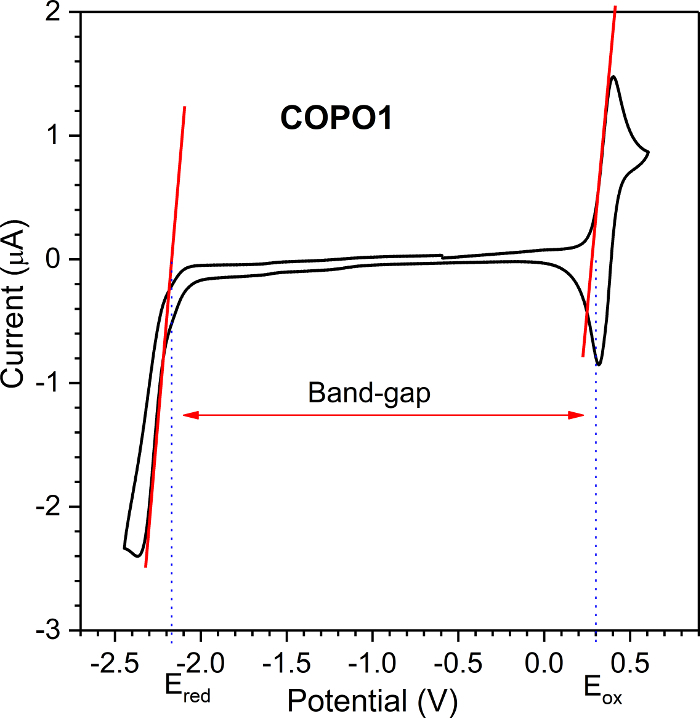

**Figure 3: Cyclic voltammetry (CV) of compound COPO1 in a wide range of potentials.** Estimation of the onset potentials for EA and IP calculations of the COPO1 compounds^52^. EA=−2.90 eV and IP = 5.35 eV. Please click here to view a larger version of this figure.

**Figure:**
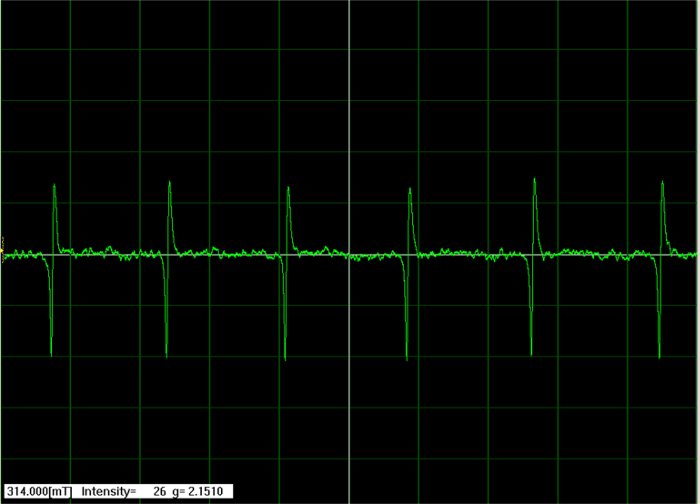

**Figure 4. EPR six spectral line of manganese standard.** The paramagnetic signal of manganese used for calibration of the signal shift. Please click here to view a larger version of this figure.

**Figure:**
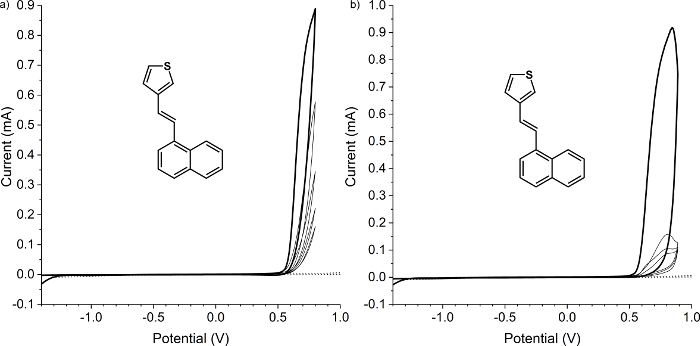

**Figure 5: Cyclic Voltammetry (CV) of compound NtVTh.** Cyclic voltammograms of 15 mM NtVTh in 0.1 M Bu_4_NBF_4_/CH_3_CN and relative to ferrocene standard presenting the degradation process involved on working electrode. Scan rate 0.05 V/s: (**a**) was taken in the range −1.4 V to 0.8 V, and (**b**) in the range −1.4 V to 0.9 V. Please click here to view a larger version of this figure.

**Figure:**
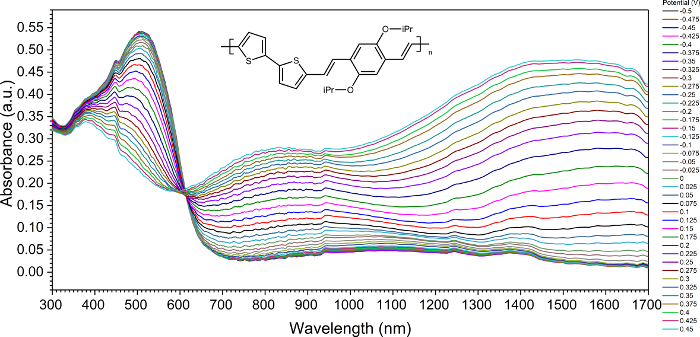

**Figure 6: Ultraviolet-visible and near-infrared (UV-Vis-NIR) spectroelectrochemistry of poly(O*i*PrThEE) derivative. **UV-Vis-NIR spectra presenting the evolution of absorption band through the generation of charge carriers on polymer structure. Please click here to view a larger version of this figure.

**Figure:**
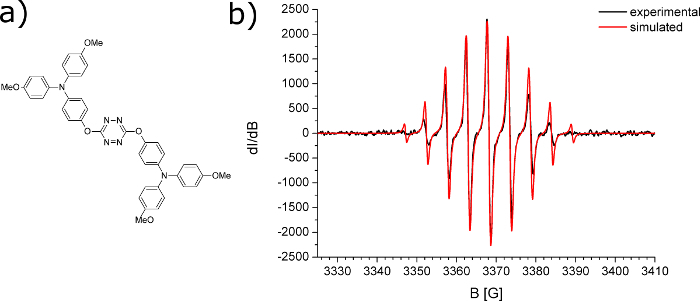

**Figure 7: EPR spectroelectrochemical analysis of tetrazine derivative.** (a) Structure of *s*-tetrazine derivative; (b) EPR spectra registered during electrochemical reduction of *s*-tetrazine derivative (black line-experimental and red line simulated spectrum). Please click here to view a larger version of this figure.

**Figure:**
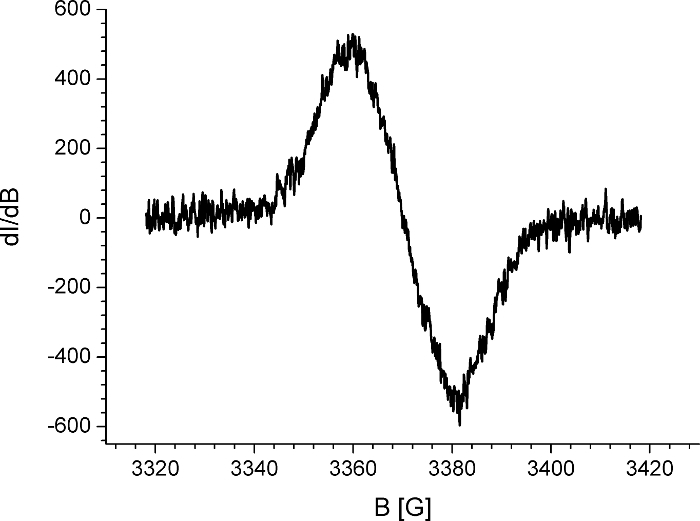

**Figure 8: EPR spectroelectrochemical polaron signal of the conjugated polymer.** Electron paramagnetic resonance (EPR) spectra registered during the first step of oxidation of conjugated polymer (EPR spectra of polaron species). Please click here to view a larger version of this figure.

**Figure:**
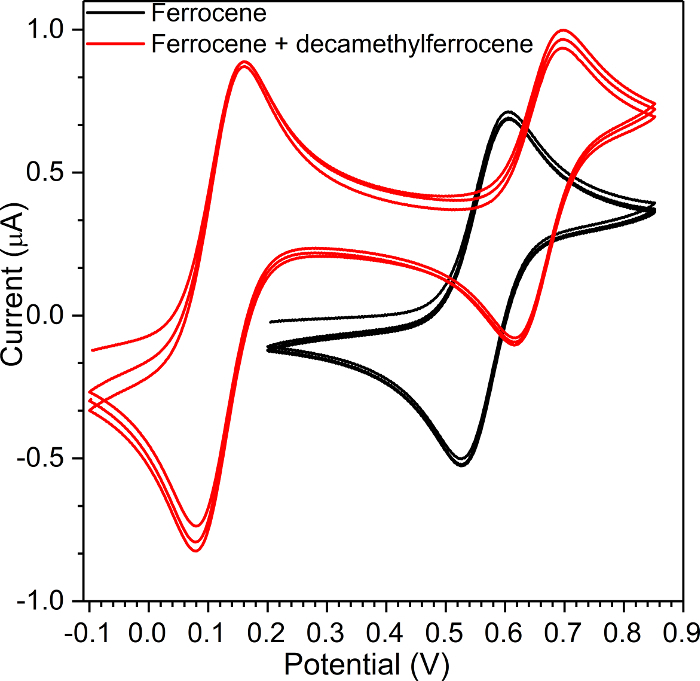

**Figure 9: Cyclic voltammetry (CV) of ferrocene and decamethylferrocene.** Comparison of two electrochemical standards as pure and as mixture showing the shift of the potential. Please click here to view a larger version of this figure.

## Discussion

Electrochemical and spectroelectrochemical techniques have no limitations; one can analyze both solid state and liquid solutions in a broad range of temperature and other conditions with these techniques. The important thing in all of these cases is that compounds/materials are analyzed under the applied potential, replicating real world conditions for working organic electronics devices. The only difference is that in electrochemistry, the formation of charge carriers, is observed.

The methods presented here show the usefulness of the analysis of charged carriers generated in organic compounds that correlate with their applicability in organic electronics. Moreover, the electrochemical and spectroelectrochemical techniques are cheaper and less demanding than that of typical methods used in charge carrier analysis, but there are some critical steps and modifications to the protocol that are needed depending on the obtained results.

During electrochemical characterization, always start with a particular concentration. If a set of the compounds is compared, then all materials need to have the same molar concentration. The best is to start with 1 mM concentration and 50 mV/s scan rate as indicated in the protocol in this study, but it is good to know the concentration of the sample on the observed electrochemical behavior. Always try to measure at least three scans. The first two scans are usually different because the starting conditions (equilibrium) are different. The second and the third scans should be the same. If the second and third scans are the same, then there are probably no side reactions observed in this system (Figure 2a). In an oxidation process, a new peak at a lower potential appears showing that the conductive material was deposited on the WE^18^,^19^,^24^,^25^,^29^,^30^,^31^,^32^. If the height of the lower peak increases in successive scans, then probably the conjugated polymer was deposited^18^,^19^,^24^,^25^,^29^,^30^,^31^,^32^. If all the currents decrease in successive scans, then the nonconductive product of degradation was deposited on the electrode. If a very small peak is observed before the main oxidation or reduction peak (especially for polymers), then this is probably charge-trapping process^19^,^23^,^31^,^34^. If a very sharp dedoping peak of oxidation or reduction is observed, then this is probably caused by the decomposition of crystalline structures on an electrode formed through the electrocrystallization process during oxidation^35^.

Always check the behavior of the test compound before, during, and after redox peaks. It means that at least three CV scans should be registered: with upper (in the case oxidation) or lower vertex potential lower or higher, respectively, then the potential of peak maximum, with upper or lower vertex potential set to exactly on the peak maximum and with vertex potentials higher (oxidation) and lower (reduction) than potential of the peak maximum. The observed process may vary and sometimes two processes may be observed under one peak theoretically. Always compare the collected cyclic voltammograms of the electrolyte (step 2.6), the ferrocene (step 2.9), the compound (step 2.13), and the ferrocene with compound (step 2.19); there are several issues to be taken into account.

Always compare the CV signals of the electrolyte and the test compound, if any signals from the electrolyte is visible on the cyclic voltammogram of the measured compound, then the electrolyte must be changed because its electrochemical window is too low, or the electrolyte is contaminated. If the signal (redox couple) of ferrocene (step 2.9) and ferrocene with compound (step 2.19) are at the same position, then everything is performed properly. If the peaks are shifted between each other, then check the RE and repeat the measurement. If the signal (oxidation, reduction, or redox couple potential) of the test compound with added ferrocene (step 2.19) is at a higher potential than that of the pure compound (step 2.13), then consider the values (oxidation, reduction, or redox couple potential) from the cyclic voltammogram of the pure compound. The shift is caused by the higher amount of ferrocene in the solution. When two oxidation processes are observed, the first process (oxidation or reduction) which is always on the WE may affect the active surface; this may cause an increase in the oxidation potential of the second process (Figure 9).

## Disclosures

The authors have nothing to disclose.
